# The evaluation of somatic cell count, microbiological quality and basic composition of sheep milk

**DOI:** 10.5194/aab-68-691-2025

**Published:** 2025-12-02

**Authors:** Jan Kuchtík, Libor Kalhotka, Květoslava Šustová, Tomáš Kopec

**Affiliations:** 1 Faculty of AgriSciences, Mendel University in Brno, Zemedelska 1, Brno 613 00, Czech Republic; 2 Department of Marketing and Tourism, AMBIS, University, Lindnerova 575/1, Praha 180 00, Czech Republic

## Abstract

The main goal of our study was to evaluate the effect of the farm, stage of lactation (SL), milking system (MS) and herd size (HS) on somatic cell count (SCC), the total count of microorganisms (TCM) and counts of selected microorganisms (lactobacilli – LBC, *Enterobacteriaceae*, enterococci, psychrotrophic microorganisms and micromycetes) in sheep milk. An additional part of our study was also the evaluation of the basic composition of milk and the pH of milk. The study was carried out on four selected sheep farms that are situated in the Czech Republic. The means of all traits found on all monitored farms were as follows: SCC – 5.31 log cells mL^−1^, TCM – 5.24 log cfu mL^−1^; LBC – 2.98 log cfu mL^−1^; *Enterobacteriaceae* – 2.31 log cfu mL^−1^; enterococci – 2.68 log cfu mL^−1^; psychrotrophic microorganisms – 4.50 log cfu mL^−1^; and micromycetes – 2.81 log cfu mL^−1^. The farm had a significant ( p≤0.05 ) effect only on SCC. The MS had a significant ( p≤0.05 ) effect on SCC and counts of LBC and psychrotrophic microorganisms. In contrast, the SL and HS had no significant effect on SCC, TCM and counts of selected microorganisms. In conclusion, it is necessary to state that all of the detected values of SCC, TCM and selected microorganisms were at a relatively very low level and were also lower than the recommended limits. This fact was, in our opinion, primarily a reflection of regular veterinary checkups and strict adherence to hygiene and sanitation standards. The results obtained are also a good prerequisite for starting the production of dairy products from unpasteurized milk on all monitored farms.

## 1 Introduction

Dairy sheep production in the Czech Republic (CR), unlike, for example, neighboring Slovakia, represents a minority in domestic livestock farming, with approximately only 2500 ewes currently being milked in the country. In the last 10 years, however, the growing interest of breeders in this production has been registered, and the numbers of dairy sheep have been slowly increasing. This gradually increasing interest of breeders was and is primarily influenced by a growth of consumer interest in fresh sheep cheese and yogurts made from this milk, with consumers of these products specifically appreciating their specific taste, as well as their natural image.

On most Czech dairy sheep farms, purebred Lacaune sheep or their crossbreeds with East Friesian sheep or Improved Wallachian sheep are reared. On some small farms, purebred ewes of Šumavská sheep are also milked. Dairy sheep farms in the CR are mainly located in foothills and mountainous areas, sheep nutrition is relatively extensive, milk production is mostly seasonal, and milk is largely processed directly on farms (Kuchtik et al., 2017).

The somatic cell count (SCC) represents an indicator of animal health status, considered to be an elective milk quality standard for liquid milk and for cheese making (Albenzio et al., 2019). The SCCs in milk vary depending on the following factors: animal species, animal, breed, stage of lactation, parity, stress, management factors, and seasonal and storage procedures (Park et al., 2013). According to Paape et al. (2007), in the United States, the limit for sheep milk is below 1 000 000 cells mL^−1^ (6.00 log), whereas there is no legal limit for this milk in the European Union. However, the threshold of SCC in sheep milk is still a debated topic. For example, Albenzio et al. (2012) proposed that the threshold of SCCs in healthy sheep mammary glands should not exceed 250 000 cells mL^−1^ (5.40 log). Nevertheless, Bianchi et al. (2004) reported the limit for subclinical mastitis in sheep to be at the level of 500 000 cells mL^−1^ (5.70 log).

The main indicator of the hygiene quality of raw sheep milk and one of the essential criteria for its purchase is the total count of microorganisms (TCM). The criteria for the hygiene quality of raw sheep milk are listed in the Regulation (EC) No. 853/2004 of the European Parliament and of the Council, as amended (Regulation 1662/2006). This regulation primarily implies that the content of microorganisms in sheep milk at 30 °C should be ≤1500000 (6.18 log) in 1 mL (moving geometric average over a 2-month period, whereby at least two samples must be taken per month). On the other hand, if the milk is intended for the production of dairy products from raw milk by a process that does not include heat treatment, the milk must contain ≤500000 (5.70 log) microorganisms in 1 mL. However, according to Gonzalo (2017), both limits are extremely high, indicative of very deficient hygiene practices, and lead to a drastic reduction in milk shelf time and a poor quality of dairy products.

In the mammary gland of a healthy animal, milk maintains a low microbial load (Fotou et al., 2011). On the other hand, it is necessary to state that raw sheep milk can contain all of the important alimentary pathogens, with *Staphylococcus aureus*, *Listeria monocytogenes*, *Escherichia coli* and *Salmonella* spp. being classified to be among these most important pathogens.

In general, the most important factors that affect the bacteriological quality of sheep milk are the hygiene of milking equipment and storage tanks, hygiene of milkers, milking method, water quality, environment in the milking parlour, temperature of the milk in the tank and hygiene of transport. According to Alexopoulos et al. (2011), other factors that can influence the bacteriological quality of sheep milk are breed, housing, season and stage of lactation.

The basic chemical composition of sheep milk can be affected by numerous factors such as the stage of lactation, parity, season, breed, environmental temperature, animal age, nutrition and diseases of the udder Claeys et al. (2014). According to Novotna et al. (2009) and Matutinovic et al. (2011), the contents of fat and total protein in sheep milk decrease until reaching the peak of milk yield, while, later, the contents of both of these milk components increase until the end of lactation. According to the same authors, the content of lactose decreases during lactation. The pH of milk has a fundamental effect on milk coagulation properties and the quality of curd (Jaramillo et al., 2008), while, according to Albenzio et al. (2004), the pH values in sheep milk increase with increasing SCCs.

In general, it can be stated that the evaluation of SCC and microbiological analyses of bulk sheep milk have been carried out only sporadically in the Czech Republic. Therefore, the main goal of our study was the evaluation of SCC, TCM and selected microorganisms in sheep milk on selected farms that are situated in the Czech Republic. The effects of stage of lactation (SL), milking system (MS) and herd size (HS) on the aforementioned traits were also evaluated as part of the study. The SCC and TCM can also affect the basic composition of milk and the pH of milk. Therefore, an integral part of our study was the evaluation of the basic components of milk and the pH of milk.

## 2 Materials and methods

### 2.1 Animal experimental design and nutrition

The study was carried out on four selected sheep farms (farms A, B, C and D) that are situated in the Czech Republic. On farm A, East Friesian sheep are reared, and on all of the other farms, Lacaune sheep are reared (Table 1). On all of the monitored farms, milk production was seasonal. On all of the farms, the lambing occurred in the course of March. Weaning of lambs on all of the farms was finished on 1 May. After weaning, all sheep on each farm began to be milked twice a day, until the end of the study. Pre-milking and post-milking disinfection was carried out for all sheep on all of the farms.

Throughout our study, all monitored farms were under regular veterinary control. At the beginning of our study, all sheep in all of the herds were clinically healthy. If symptoms of mastitis were detected in individual ewes on any of the farms during the study, these ewes were removed from production.

Throughout the monitored period, the sheep on all of the farms were kept on all-day pastures, with the daily feed rations of ewe's on all of these farms being very similar as they consisted of pasture (ad libitum), concentrate mixture (approx. 1 kg d^−1^ per ewe) and mineral lick (ad libitum). In the case of rains that lasted 2 or more days, the hay was added (ad libitum) to the sheep diet on all of the farms.

During our study, all ewes on the individual farms were kept in one flock under identical conditions without any discernible differences in their nutrition or management. After pasteurization, milk on all of the farms was processed into various dairy products, while the main product on all of the farms was fresh sheep cheese. Other specific characteristics of the individual farms are shown in Table 1.

**Table 1:** Other specific characteristics of the individual farms.

Farms	A	B	C	D
Farming system	Conventional	Organic	Conventional	Conventional
Breed	East Friesian	Lacaune	Lacaune	Lacaune
Average milk production per sheep and lactation	436	271	285	308
Herd size	17	110	210	48
Milking	Machine	Machine	Machine	Machine
Milking system	Bucket	Bucket	Pipeline	Pipeline
Milk cooling	Glass containers in the refrigerator	Stainless steel cooling tank	Stainless steel cooling tank	Stainless steel cooling tank
Other farm activities	Horse breeding and beekeeping	Agro-tourism and beekeeping	Restaurant	Breeding of suckler cows

### 2.2 Samplings

All samplings were carried out from bulk milk during 2023. All samples from all of the samplings contained milk from the previous evening and morning milking. The first sampling was carried out in May, the second was carried out in July, and the last was carried out in September. These periods correspond to the three stages of production (May is the early stage of lactation, July is the middle stage of lactation, and September is the late stage of lactation). Within the framework of the first sampling, samplings were taken on all four farms, with these samplings being obtained from the discharge valve of the tank (after thorough mixing beforehand) in two sterile containers and one small sterile sample container. The milk from the first sterile container was used for analyses of the basic milk components and pH. Milk from the second sterile container was used for bacteriological analyses. Milk from the small sterile sample container (each containing one Broad Spectrum Microtabs preservative tablet) was used for the determination of SCC. In the same way, two more samplings (in July and September) were carried out on all farms. Finally, it should be added that a total of nine samplings were carried out on each farm during our entire study.

After samplings, all of the individual milk samples were transported in cooling boxes at a temperature of about 5 °C to the milk laboratories at Mendel University in Brno and to the private Laboratory for Milk Analysis in Brno – Tuřany (Bohemian-Moravian Association of Breeders, a.s.).

### 2.3 Milk analysis

The SCC was determined using the fluoro-opto-electronic apparatus BENTLEY 2500 (Czech State Standard EN ISO No. 13366-2:2006). The total count of microorganisms (TCM) was determined according to the Czech State Standard ISO 6610:1992. The count of lactic acid bacteria (lactobacilli – LBC) was determined on MRS medium (Biokar Diagnostics, France) anaerobic cultivation at 37 °C for 48 h (Kalhotka et al., 2015). The count of *Enterobacteriaceae* was determined according to the Czech State Standard ISO 7402:1995. The count of enterococci was determined on Slanetz-Bartley Agar (Merck, Germany) at 37 °C 48 h (Bogdanovicova et al., 2016). The count of psychrotrophic microorganisms was determined according to the Czech State Standard ISO 6730:1996. The count of micromycetes – yeast and molds – was determined according to the Czech State Standard ISO 7954:1994. Afterwards, typical colonies were counted, and the result was expressed as cfu (colony-forming units) per mL.

Fat (F) content (in %) was determined by Gerber's acidobutyrometric method (Czech Technical Standard ISO No. 2446:2008). Total protein (TP) content (in %) was determined according to the Czech Technical Standard EN ISO 8968-1:2014 using a Kjeltec (Foss Electric, Denmark). Lactose (L) content (in %) was determined polarimetrically (Czech Technical Standard No. 570530:1979). Active acidity (pH) was measured with the pH meter WTW 95 with the probe WTW SenTix 97.

### 2.4 Statistical analysis

First, the counts of SCC and TCM and the counts of Lactobacilli, *Enterobacteriaceae*, Enterococci, psychrotrophic bacteria and micromycetes were transformed into a logarithmic form to normalize their frequency distribution before performing statistical analysis. A statistical analysis was carried out using STATISTICA CZ version 14. ANOVA analysis was applied to evaluate the differences between the individual farms, stages of lactation, milking systems and herd sizes. When the analysis of variance showed significant differences between the groups, Tukey's test was applied. The differences were considered to be significant if p≤0.05 . Pearson correlation coefficients were also calculated using Statistica 14.

**Table 2:** Effect of the farm on SCC (log cells mL^−1^), TCM (log cfu mL^−1^) and counts of selected microorganisms (log cfu mL^−1^).

Traits	Farm A				Farm B				Farm C				Farm D				All farms			
	Mean	SD	Min.	Max.	Mean	SD	Min.	Max.	Mean	SD	Min.	Max.	Mean	SD	Min.	Max.	Mean	SD	Min.	Max.
SCC	5.02^b^	0.31	4.84	5.38	5.35^a,b^	0.06	5.28	5.41	5.37^a,b^	0.06	5.32	5.43	5.48^a^	0.03	5.44	5.51	5.31	0.22	4.84	5.51
TCM	4.99	0.50	4.54	5.52	5.53	0.40	5.07	5.80	5.27	0.52	4.70	5.73	5.17	0.28	4.88	5.44	5.24	0.42	4.54	5.80
LBC	3.09	0.60	2.69	3.77	3.51	1.09	2.31	4.41	2.28	0.51	1.70	2.61	3.03	0.18	2.92	3.23	2.98	0.74	1.70	4.41
*Entb.*	1.44	0.48	1.15	2.00	2.56	0.78	1.74	3.29	2.55	0.95	2.00	3.65	2.68	0.32	2.34	2.97	2.31	0.78	1.15	3.65
Entc.	2.64	0.84	1.70	3.30	2.81	0.68	2.13	3.50	2.35	0.34	2.06	2.72	2.92	0.46	2.63	3.45	2.68	0.57	1.70	3.50
Psych.	5.00	0.48	4.45	5.31	4.92	0.64	4.33	5.60	3.94	0.31	3.60	4.22	4.15	0.36	3.80	4.52	4.50	0.63	3.60	5.60
Micrm.	2.10	1.00	1.40	3.25	3.02	0.92	2.02	3.82	2.92	0.23	2.71	3.16	3.21	0.52	2.69	3.72	2.81	0.77	1.40	3.82

SD: standard deviation; min.: minimum; max.: maximum. ^a,b^ Means with different superscripts differ at p≤0.05 . SCC: somatic cell count; TCM: total count of microorganisms; LBC: lactobacilli; *Entb.*: *Enterobactericeae*; Entc.: Enterococci; Psych.: psychrotrophic microorganisms; Microm.: micromycetes; cfu: colony-forming units.

## 3 Results

### 3.1 Effect of farm, stage of lactation, milking system and herd size on somatic cell count, total count of microorganisms and counts of selected microorganisms

The means (Table 2) of all logarithms found on all of the monitored farms were as follows: SCC – 5.31 log cells mL^−1^; TCM – 5.24 log cfu mL^−1^; LBC – 2.98 log cfu mL^−1^; *Enterobacteriaceae* – 2.31 log cfu mL^−1^; enterococci – 2.68 log cfu mL^−1^; psychrotrophic microorganisms – 4.50 log cfu mL^−1^; and micromycetes – 2.81 log cfu mL^−1^. A significant ( p≤0.05 ) effect of the farm (Table 2) was found only on log SCC, with the significantly highest log SCC (5.48) being found on farm D. In contrast, the significantly lowest log SCC (5.02) was found on farm A.

**Table 3:** Effect of the stage of lactation on SCC (log cells mL^−1^), TCM (log cfu mL^−1^) and counts of selected microorganisms (log cfu mL^−1^).

Traits	Early stage of lactation				Middle stage of lactation				Late stage of lactation				All stages of lactation			
	Mean	SD	Min.	Max.	Mean	SD	Min.	Max.	Mean	SD	Min.	Max.	Mean	SD	Min.	Max.
SCC	5.23	0.27	4.84	5.44	5.26	0.27	4.86	5.48	5.43	0.05	5.38	5.51	5.31	0.22	4.84	5.51
TCM	5.28	0.37	4.89	5.73	5.06	0.56	4.54	5.80	5.38	0.35	4.88	5.71	5.24	0.42	4.54	5.80
LBC	2.69	0.39	2.31	3.23	3.19	0.83	2.61	4.41	3.05	0.99	1.70	3.81	2.98	0.74	1.70	4.41
*Enterobacteriaceae*	2.02	0.25	1.74	2.34	2.13	0.73	1.15	2.74	2.77	1.10	1.18	3.65	2.31	0.76	1.15	3.65
Enterococci	2.18	0.39	1.70	2.63	2.78	0.12	2.67	2.93	3.08	0.69	2.06	3.50	2.68	0.57	1.70	3.50
Psychrotrophic m.	4.26	0.76	3.60	5.31	4.59	0.68	4.11	5.60	4.65	0.52	4.00	5.24	4.50	0.63	3.60	5.60
Micromycetes	3.02	0.27	2.69	3.25	2.64	0.97	1.65	3.72	2.78	1.03	1.40	3.82	2.81	0.77	1.40	3.82

SD: standard deviation; min.: minimum; max.: maximum; SCC: somatic cell count; TCM: total count of microorganisms; LBC: lactobacilli; cfu: colony-forming units.

**Table 4:** Effect of the milking system on SCC (log cells mL^−1^), TCM (log cfu mL^−1^) and counts of selected microorganisms (log cfu mL^−1^).

Traits	Bucket				Pipeline				All milking systems			
	Mean	SD	Min.	Max.	Mean	SD	Min.	Max.	Mean	SD	Min.	Max.
SCC	5.19^b^	0.27	4.84	5.41	5.42^a^	0.07	5.32	5.51	5.31	0.22	4.84	5.51
TCM	5.26	0.50	4.54	5.80	5.22	0.38	4.70	5.73	5.24	0.42	4.54	5.80
LBC	3.30^a^	0.82	2.31	4.41	2.66^b^	0.53	1.70	3.23	2.98	0.74	1.70	4.41
*Enterobacteriaceae*	2.00	0.84	1.15	3.29	2.62	0.64	2.00	3.65	2.31	0.78	1.15	3.65
Enterococci	2.73	0.69	1.70	3.50	2.63	0.48	2.06	3.45	2.68	0.57	1.70	3.50
Psychrotrophic m.	4.96^a^	0.51	4.33	5.60	4.04^b^	0.32	3.60	4.52	4.50	0.63	3.60	5.60
Micromycetes	2.56	1.00	1.40	3.82	3.07	0.39	2.69	3.72	2.81	0.77	1.40	3.82

SD: standard deviation; min.: minimum; max.: maximum. ^a,b^ Means with different superscripts differ at p≤0.05 . SCC: somatic cell count; TCM: total count of microorganisms; LBC: lactobacilli; cfu: colony-forming units.

**Table 5:** Effect of the herd size on SCC (log cells mL^−1^), TCM (log cfu mL^−1^) and counts of selected microorganisms (log cfu mL^−1^).

Traits	Up to 100 ewes				More than 100 ewes				All herd sizes			
	Mean	SD	Min.	Max.	Mean	SD	Min.	Max.	Mean	SD	Min.	Max.
SCC	5.25	0.31	4.84	5.51	5.36	0.06	5.28	5.43	5.31	0.22	4.84	5.51
TCM	5.08	0.37	4.54	5.52	5.40	0.44	4.70	5.80	5.24	0.42	4.54	5.80
LBC	3.06	0.39	2.69	3.77	2.90	1.01	1.70	4.41	2.98	0.74	1.70	4.41
*Enterobacteriaceae*	2.06	0.77	1.15	2.97	2.55	0.78	1.74	3.65	2.31	0.78	1.15	3.65
Enterococci	2.78	0.62	1.70	3.45	2.58	0.55	2.06	3.50	2.68	0.57	1.70	3.50
Psychrotrophic m.	4.57	0.60	3.80	5.31	4.43	0.70	3.60	5.60	4.50	0.63	3.60	5.60
Micromycetes	2.66	0.94	1.40	3.72	2.97	0.60	2.02	3.82	2.81	0.77	1.40	3.82

SD: standard deviation; min.: minimum; max.: maximum; SCC: somatic cell count; TCM: total count of microorganisms; LBC: lactobacilli; cfu: colony-forming units.

The stage of lactation (SL) had no significant effect on SCC, TCM and counts of selected microorganisms (Table 3). Milking system (MS) had a significant ( p≤0.05 ) effect on log SCC, log LBC and log of psychrotrophic microorganisms (Table 4), while lower values of log LBC (2.66) and log of psychrotrophic microorganisms (4.04) were found in the case of the MS with a pipeline compared to the MS with a bucket (3.30 and 4.96). On the contrary, lower log SCC was found in the case of the MS with a bucket compared to the MS with a pipeline (5.19 vs. 5.42). The herd size (HS) had no significant effect on SCC, TCM and counts of selected microorganisms (Table 5).

**Table 6:** Effect of the farm on the basic composition of sheep milk and its pH.

Traits	Farm A				Farm B				Farm C				Farm D				All farms			
	Mean	SD	Min.	Max.	Mean	SD	Min.	Max.	Mean	SD	Min.	Max.	Mean	SD	Min.	Max.	Mean	SD	Min.	Max.
Fat (%)	5.72^b^	1.03	5.01	6.90	7.66^a^	0.63	6.94	8.10	6.98^a^	0.90	6.20	7.97	6.64^a,b^	1.28	5.33	7.88	6.75	1.12	5.01	8.10
TP (%)	5.59^a,b^	1.00	4.76	6.54	5.57^a,b^	0.58	5.03	6.18	5.48^b^	0.58	5.14	6.15	6.97^a^	1.76	5.41	8.88	5.85	1.15	4.76	8.88
Lactose (%)	4.71	0.53	4.13	5.17	4.71	0.31	4.47	5.06	4.52	0.36	4.11	4.77	4.49	0.41	4.12	4.93	4.61	0.37	4.11	5.17
pH	6.57	0.03	6.55	6.60	6.51	0.05	6.46	6.55	6.61	0.13	6.47	6.72	6.62	0.12	6.52	6.75	6.58	0.09	6.46	6.75

TP: total protein; SD: standard deviation; min.: minimum; max.: maximum. ^a,b^ Means shown with different superscripts differ at p≤0.05 .

**Table 7:** Effect of the stage of lactation on the basic composition of sheep milk and its pH.

Traits	Early stage of lactation				Middle stage of lactation				Late stage of lactation				All stages of lactation			
	Mean	SD	Min.	Max.	Mean	SD	Min.	Max.	Mean	SD	Min.	Max.	Mean	SD	Min.	Max.
Fat (%)	5.93	0.80	5.24	6.94	6.62	1.21	5.01	7.95	7.71	0.55	6.90	8.10	6.75	1.12	5.01	8.10
TP (%)	5.09^b^	0.27	4.76	5.41	5.53^a,b^	0.77	4.86	6.61	6.94^a^	1.31	6.15	8.88	5.85	1.15	4.76	8.88
Lactose (%)	4.98^a^	0.17	4.77	5.17	4.63^b^	0.17	4.41	4.82	4.21^c^	0.18	4.11	4.47	4.61	0.37	4.11	5.17
pH	6.60	0.09	6.52	6.72	6.56	0.07	6.46	6.63	6.57	0.12	6.47	6.75	6.58	0.09	6.46	6.75

TP: total protein; SD: standard deviation; min.: minimum; max.: maximum. ^a,b^ Means with different superscripts differ at p≤0.05 .

**Table 8:** Effect of the milking system on the basic composition of sheep milk and its pH.

Traits	Bucket				Pipeline				All milking systems			
	Mean	SD	Min.	Max.	Mean	SD	Min.	Max.	Mean	SD	Min.	Max.
Fat (%)	6.69	1.31	5.01	8.10	6.81	1.01	5.33	7.97	6.75	1.12	5.01	8.10
TP (%)	5.48	0.74	4.76	6.54	6.22	1.43	5.14	8.88	5.85	1.15	4.76	8.88
Lactose (%)	4.71	0.39	4.13	5.17	4.51	0.35	4.11	4.93	4.61	0.37	4.11	5.17
pH	6.54	0.05	6.46	6.60	6.61	0.11	6.47	6.75	6.58	0.09	6.46	6.75

TP: total protein; SD: standard deviation; min.: minimum; max.: maximum.

**Table 9:** Effect of the herd size on the basic composition of sheep milk and its pH.

Traits	Up to 100 ewes				More than 100 ewes				All herd sizes			
	Mean	SD	Min.	Max.	Mean	SD	Min.	Max.	Mean	SD	Min.	Max.
Fat (%)	6.18^b^	1.16	5.01	7.88	7.32^a^	0.79	6.20	8.10	6.75	1.12	5.01	8.10
TP (%)	6.18	1.55	4.76	8.88	5.52	0.52	5.03	6.18	5.85	1.15	4.76	8.88
Lactose (%)	4.60	0.44	4.12	5.17	4.62	0.32	4.11	5.06	4.61	0.37	4.11	5.17
pH	6.59	0.08	6.52	6.75	6.56	0.10	6.46	6.72	6.58	0.09	6.46	6.75

TP: total protein; SD: standard deviation; min.: minimum; max.: maximum. ^a,b^ Means with different superscripts differ at p≤0.05 .

### 3.2 Effect of farm, stage of lactation, milking system and herd size on basic composition of sheep milk and its pH

The mean contents of all basic components of sheep milk found on all of the monitored farms were as follows: F – 6.75 %; TP – 5.85 %; and L – 4.61 %. The overall mean of the pH was 6.58. The farm (Table 6) had a significant ( p≤0.05 ) effect on the contents of F and TP. The highest fat contents were found on farms B and C (7.66 % and 6.98 %), while the lowest content of this milk component was found on farm A (5.72 %). The significantly highest content of TP (6.97 %) was found on farm D. On the contrary, the lowest content (5.48 %) was found on farm C. The SL (Table 7) had a significant ( p≤0.05 ) effect on the contents of TP and L. The lowest content of TP (5.09 %) was found in the early SL, and its highest content (6.94 %) was found in the late SL. The highest content of L (4.98 %) was found in the early SL, and its lowest content (4.21 %) was found in the late SL. The milking system (MS) had no significant effect on the basic composition of sheep milk and its pH (Table 8). The HS (Table 9) had a significant ( p≤0.05 ) effect only on F content when its highest value (7.32 %) was found in the case of HS with more than 100 heads.

**Table 10:** Correlation between all monitored traits.

	Fat	Total protein	Lactose	pH	Log SCC	Log TCM	Log LBC	Log *Enterobac.*	Log enterococci	Log psychro.	Log micromyc.
Fat	1.00	0.57	-0.69 *	-0.16	0.68*	0.44	0.20	0.67*	0.39	0.21	0.22
Total protein	0.57	1.00	-0.79 *	0.35	0.60*	0.04	0.13	0.46	0.60*	0.04	0.16
Lactose	-0.69 *	-0.79 *	1.00	-0.01	-0.62 *	-0.25	-0.12	-0.45	-0.57	-0.05	0.13
pH	-0.16	0.35	-0.01	1.00	0.02	-0.35	-0.28	-0.18	0.08	-0.31	0.27
Log SCC	0.68*	0.60*	-0.62 *	0.02	1.00	0.55	0.16	0.56	0.38	-0.31	0.25
Log TCM	0.44	0.04	-0.25	-0.35	0.55	1.00	0.47	0.36	0.11	0.09	-0.05
Log LBC	0.20	0.13	-0.12	-0.28	0.16	0.47	1.00	-0.07	0.62*	0.65*	-0.29
Log *Enterobac.*	0.67*	0.46	-0.45	-0.18	0.56	0.36	-0.07	1.00	0.05	-0.14	0.56
Log enterococci	0.39	0.60*	-0.57	0.08	0.38	0.11	0.62*	0.05	1.00	0.22	-0.16
Log psychrotro.	0.21	0.04	-0.05	-0.31	-0.31	0.09	0.65*	-0.14	0.22	1.00	-0.31
Log micromyc.	0.22	0.16	0.13	0.27	0.25	-0.05	-0.29	0.56	-0.16	-0.31	1.00

SCC: somatic cell count; TCM: total count of microorganisms; LBC: lactobacilli; *Enterobac*.: *Enterobacteriaceae*; psychro.: psychrotrophic microorganisms; micromyc.: micromycetes; * p≤0.05 .

### 3.3 Correlations between all of the monitored traits

The correlations between all of the monitored traits are presented in Table 10. The F content had a significant ( p≤0.05 ) positive correlation with log SCC (0.68) and log *Enterobacteriaceae* (0.67) and a negative significant ( p≤0.05 ) correlation ( -0.69 ) with the content of L. The TP content had a significant ( p≤0.05 ) positive correlation with log SCC (0.60) and log enterococci (0.60) and a negative significant ( p≤0.05 ) correlation ( -0.79 ) with the content of L. The L content had also a significant ( p≤0.05 ) negative correlation (–0.79) with log SCC. The log SCC had no other significant correlation apart from the above. The log TCM did not have a significant correlation with any of the monitored traits. The log LBC had a significant ( p≤0.05 ) positive correlation only with log enterococci (0.62) and log psychrotrophic microorganisms (0.65). The log *Enterobacteriaceae*, the log enterococci and the log psychrotrophic microorganisms did not have any other significant correlation apart from the significant correlations mentioned above. The log micromycetes did not have a significant correlation with any of the monitored traits.

## 4 Discussion

### 4.1 Effect of farm, stage of lactation, milking system and herd size on somatic cell count

Somatic cells are a reflection of the inflammatory response to an intra-mammary infection or another trigger of the immune system or of the physiological phase of lactation (Albenzio et al., 2019). In our opinion, the fact that all of the monitored farms in our study were under regular veterinary control when all ewes with symptoms of mastitis were removed from production had a favorable effect on the low values of log SCC within all of the factors we monitored (Tables 2, 3, 4 and 5). All of the detected values of SCC were also significantly lower than the limits published by Bianchi et al. (2004) and Paape et al. (2007) and the data published by Carloni et al. (2016).

However, the fact is that our study showed a significant ( p≤0.05 ) effect of farm and MS on the log of SCC, when the lowest values of the log of SCC were found on farm A and in the MS with a bucket. In our opinion, the lowest value of log SCC on farm A was mainly affected by the lowest number of ewes reared here as this situation allowed the breeder to have enough time for individual care of each sheep and a better individual approach to milking. Regarding the lower logarithms of SCC in the MS with a bucket, we assume that this was a consequence of lower SCCs on both farms (A and B), where the MS with a bucket was applied, compared to the both farms (C and D) where the MS with a pipeline was applied. However, in our opinion, the MS should have a minimal effect on SCC.

Many studies show – see, for example, Paape et al. (2007) and Matutinovic et al. (2011) – that the SCC increases significantly during lactation. In our study, the SCC grew non-significantly during lactation, which is, however, in accordance with results published by Jaramillo et al. (2008) and Kuchtik et al. (2017). In our opinion, the insignificant increase in SCC recorded during lactation in our study could have been affected by the reduction in milk yield during lactation and also, as reported Sevi et al. (1999), by a functional alteration of the mammary gland tissue caused by shorter survival of epithelial cells that are supplied with fewer nutrients at the end of lactation.

In conclusion for this part, it should be added that the significant ( p≤0.05 ) negative correlation between the SCC and L content found in our study is quite interesting as it demonstrates that the decreasing L content is a reflection of the increasing SCC. We assume that this correlation also suggests that the determination of L content can be a useful tool to aid in mastitis detection.

### 4.2 Effect of farm, stage of lactation, milking system and herd size on total counts of microorganisms and counts of selected microorganisms in sheep milk

The TCM is an essential criterion for the microbiological quality of milk, while this quality is primarily influenced by the milking method, breed, housing, season, stage of lactation and farm hygiene (Alexopoulos et al., 2011). In our study, the farm did not have a significant effect on TCM, with its values, depending on the farm, ranging from 4.54 to 5.80 log cfu mL^−1^. These values are significantly lower than the criteria presented in the Regulation (EC) No. 853/2004 for sheep milk and the values found in sheep milk by Kondyli et al. (2012) and Tonamo et al. (2020). On the other hand, however, the values found in our study are comparable to the results published by Carloni et al. (2016). Likewise, other factors such as SL, MT and HS did not have a significant effect on TCM, while, in the case of all of the above-mentioned factors, the TCM values were also lower than the criteria presented in the Regulation (EC) No. 853/2004 for sheep milk.

Regarding the individual correlations between TCM and all of the other traits, the relatively high but insignificant correlation between TCM and SCC (0.55) appears to be quite interesting since a similarly high correlation between the same traits in sheep milk was also reported by de Garnica et al. (2013), while Koop et al. (2009) found a significant correlation between these traits in goat milk. However, in our opinion, the increase in SCCs in the milk of healthy ewes in our study may not have a significant effect on TCMs and vice versa since the SCCs in healthy ewes are primarily affected by the stage of lactation, whereas the TCM is primarily influenced by milking hygiene. In conclusion to the above, it is necessary to add that the TCM also had no significant correlation with any of the other monitored parameters.

A common microflora of raw milk includes lactic acid bacteria (Lactobacilli). These microorganisms get into the milk mainly from the surface of the udders or from the teats but also from the feed. In our study, the counts of LAB (respectively, Lactobacilli – LBC) ranged from 1.70 to 4.41 log cfu mL^−1^, with an average for all of the farms at the level of 2.98 log cfu mL^−1^, with these being significantly lower logarithms than reported Kondyli et al. (2012) and Silva et al. (2020) for these microorganisms.

Regarding the individual factors, the farm, SL and HS did not have a significant effect on the counts of LBC. In contrast, the MS had a significant ( p≤0.05 ) effect on this trait, with a lower count of these microorganisms being detected in the case of MS by pipeline. This fact was mainly influenced by the lower counts of LBC on both the farms where MS by pipeline was applied. Regarding individual correlations, in this case, a significant ( p≤0.05 ) positive correlation was found between log LBC and log enterococci and between log LBC and log psychrotrophic microorganisms. The above can be explained by the fact that enterococci belong to lactic acid bacteria, as well as LBC (Franz et al., 2003), and that some types of LBC can also be psychrotrophic microorganisms (Samarzija et al., 2012).

An important group of bacteria that also contaminate raw milk are bacteria of the family *Enterobacteriaceae*. Most *Enterobacteriaceae* live in the digestive tract of vertebrates as a natural part of the intestinal microflora. Most of these bacteria are non-pathogenic, but some (e.g., *Salmonella, Shigella*, some strains of *E. coli*) can be the cause of serious and fatal diseases for humans. Since enterobacteria living in the intestine enter the external environment through faeces, their presence (for example, in water) indicates faecal pollution. However, for raw sheep milk, there is no limit for the contents of these bacteria in the Czech Republic.

The counts of *Enterobacteriaceae* in our study ranged from 1.15 to 3.65 log cfu mL^−1^, with none of the monitored factors having a significant effect on this trait. The detected counts of *Enterobacteriaceae* were comparable to the values reported by Kondyli et al. (2012) and Merlin Junior et al. (2015). In contrast, Tonamo et al. (2020) reported higher values of this log, while, in their opinion, these higher values were influenced by the level of hygiene and sanitation and the technological level in obtaining and storing milk. The counts of *Enterobacteriaceae* had only one significant ( p≤0.05 ) correlation (Table 10), namely with the content of F. However, we have no explanation for this correlation.

Enterococci are lactic acid bacteria (LAB) that are important in environmental, food and clinical microbiology (Franz et al., 2003). Enterococci represent a large part of the autochthonous bacteria associated with the mammalian gastrointestinal tract but are also often found in soil and water and on plants (Švec and Franz, 2014). Enterococci also commonly contaminate raw and pasteurized milk, and their presence in milk and milk products is indicative of insufficient sanitary conditions during milk acquisition and processing (Lebreton et al., 2014; Lacanin et al., 2015). However, Greifova et al. (2003) state that milk can also be contaminated with Enterococci from feed.

In our study, the counts of enterococci ranged from 1.70 to 3.50 log cfu mL^−1^, with an average for all of the farms at the level of 2.68 log cfu mL^−1^, which are values comparable to the data reported for sheep milk by Kondyli et al. (2012). In contrast, Duckova et al. (2008) found significantly higher counts of this bacteria in sheep milk. Anyway, in relation to the individual factors, the logarithms of this trait were very balanced, and none of the monitored factors had a significant effect on this trait.

Psychrotrophic microorganisms are among the most important contaminants of sheep milk. They make up more than 90 % of the microbial population of chilled milk (Silanikove et al., 2010). These microorganisms are also very important as they are capable of growth and metabolic manifestations even at low temperatures, which can be very problematic if milk is stored in a tank at 4 °C for more than 24 h. In our study, the counts of these microorganisms related to all of the evaluated factors were also dominant. Regarding the influence of the individual factors on this trait, the factors of the farm, SL and HS did not have a significant effect. On the contrary, a significant ( p≤0.05 ) effect of MS on this trait was found, with lower counts of these microorganisms being recorded on both farms (C and D) where the MS a with pipeline was applied. In our opinion, lower counts of these microorganisms in the case of the MS with a pipeline are a reflection of the fact that milk, when using this system, is directly transported under vacuum from the udder to dairy for cooling and storage. At the same time, this system is characterized by a significant reduction in environmental contamination, whereas the MS with a pipeline is more suitable in terms of both hygiene and sanitation compared to the MS with a bucket.

Although the SL did not have a significant effect on this trait, a gradual increase in the count of these microorganisms during lactation was registered in our study, with, for example, de Garnica et al. (2013) also recording an increase in the counts of these microorganisms from spring to winter, with the exception being during the summer period.

Raw sheep milk also contains microscopic fungi (micromycetes – yeasts and molds). Representatives of the genera *Candida, Cryptococcus, Debaryomyces, Geotrichum, Kluyveromyces, Malassezia, Pichia, Rhodotorula, Trichosporon, Aspergillus, Chrysosporium, Cladosporium, Engyodontium, Fusarium, Penicillium* and *Torrubiella* are usually detected in raw milk (Delavenne et al., 2011). The most important sources of micromycetes in milk are the udder surface, feed and the surrounding environment. In our study, the counts of micromycetes ranged from 1.40 to 3.82 log cfu mL^−1^ depending on the individual farm, with the mean for all of the farms being at the level of 2.81 log cfu mL^−1^. The above-mentioned range is comparable to the range reported by Kondyli et al. (2012). In conclusion to the above, it must be stated that the counts of the micromycetes were not affected by any of the monitored factors, and this trait also had no significant correlation with the other monitored traits.

### 4.3 Effect of farm, stage of lactation, milking system and herd size on basic milk composition and pH

The farm had a significant ( p≤0.05 ) effect on the contents of F and TP (Table 6). In our opinion, the effect on the content of F was mainly affected by the negative correlation between milk yield and F content because East Friesian (EF) sheep which were reared on farm A had a significantly higher milk yield than the sheep of the Lacaune breed which were reared on all of the other farms. However, the contents of F on most of the monitored farms were higher than the contents of this milk component reported by Khaldi et al. (2022) but were comparable to the data reported by Carloni et al. (2016). In contrast, the contents of TP were quite balanced both on farm A, where EF sheep were reared, and on farms B and C, where Lacaune sheep were reared. However, the significantly ( p≤0.05 ) highest content of TP was found on farm D, where Lacaune sheep were also reared. In our opinion, this fact was probably influenced by the long-term breeding of sheep on this farm for the content of protein in the milk. However, the mean content of TP for all of the farms was comparable to the data found by Carloni et al. (2016) and was slightly lower than that reported by Kondyli et al. (2012). The contents of L were quite balanced on all farms, and their values were comparable to the data published in reviews by Claeys et al. (2014) and Balthazar et al. (2017). The pH values on the all farms were also very balanced, and all values of this trait were comparable to the data reported by Mayer and Fiechter (2012) and Li et al. (2022).

The SL had a significant ( p≤0.05 ) effect on the contents of TP and L (Table 7). The contents of F and TP increased during lactation, which is in line with the trends reported by Kuchtik et al. (2017), Li et al. (2022), and Antunović et al. (2024). On the contrary, the content of L decreased significantly ( p≤0.05 ) during lactation, which is in accordance with the trends published by Li et al. (2022) and Antunović et al. (2024). The above trends are also confirmed in our study by the correlations between L and F and L and TP (Table 10), with both of these correlations being significantly ( p≤0.05 ) negative.

The pH of sheep milk was quite balanced during the whole lactation period, and the SL had no significant effect on this trait, which is in line with the findings of Albenzio et al. (2019).

The HS (Table 9) had a significant ( p≤0.05 ) effect only on the F content, with higher values being found on the farms with more than 100 ewes. In our opinion, the significantly higher F content on farms with more than 100 ewes was mainly influenced by the fact that on both of these farms reared Lacaune ewes, which usually have a higher F content than EF ewes that were reared on one of the smaller farms.

## 5 Conclusions

The results of our study mainly showed that the farm had a significant effect on the somatic cell count, and the milking system had a significant effect on somatic cell counts and counts of lactobacilli and psychrotrophic microorganisms. Contrawise, the stage of lactation and herd size had no significant effect on somatic cell count and microbiological traits. At the same time, it is necessary to state that all of the detected values of somatic cell count and the total counts of microorganisms were at a relatively very low level and were also lower than the recommended limits. This fact was, in our opinion, primarily a reflection of regular veterinary checkups that were carried out on all of the farms and strict adherence to hygiene and sanitation standards. At the very end, it should be added that the results obtained are a good prerequisite for the monitored farms to start producing sheep dairy products from unpasteurized milk, with the demand for these products having been increasing significantly in recent years.

## Data Availability

The data presented in this study are available on request from the corresponding author.

## References

[bib1.bib1] Albenzio M, Caroprese M, Marino R, Santillo A, Taibi L,  Sevi A (2004). Effects of somatic cell count and stage of lactation on the plasmin activity and cheese-making properties of ewe milk. J Dairy Sci.

[bib1.bib2] Albenzio M, Caroprese M, Santillo A, Ruggieri D, Ciliberti MG, Sevi A (2012). Immune competence of the mammary gland as affected by somatic cell and pathogenic bacteria in ewes with subclinical mastitis. J Dairy Sci.

[bib1.bib3] Albenzio M, Figliola L, Caroprese M, Marino R, Sevi A, Santillo A (2019). Somatic cell count in sheep milk. Small Rum Res.

[bib1.bib4] Alexopoulos A, Tzatzimakis G, Bezirtzoglou E, Plessas S, Stavropoulou E, Sinapis E, Abas Z (2011). Microbiological quality and related factors of sheep milk produced in farms of NE Greece. Anaerobe.

[bib1.bib5] Antunović Z, Mioč B, Klir Šalavardić Ž, Širić I, Držaić V, Mikulec N, Krivohlavek A, Novoselec J (2024). Physical and chemical properties, hygienic quality and fatty acid profile in milk of lactating Lacaune dairy sheep. Arch Anim Breed.

[bib1.bib6] Balthazar CF, Pimentel TC, Ferrão LL, Almada CN, Santillo A, Albenzio M, Mollakhalili N, Mortazavian AM, Nascimento JS, Silva MC, Freitas MQ, Sant'Ana AS, Granato D, Cruz AG (2017). Sheep Milk: Physicochemical Characteristics and Relevance for Functional Food Development. Compr Rev Food Sci Food Saf.

[bib1.bib7] Bianchi L, Bolla A, Budelli E, Carovi A, Fazoli C, Pauselli M, Duranti E (2004). Effect of udder health status and lactation phase on the characteristics of Sardinian ewe milk. J Dairy Sci.

[bib1.bib8] Bogdanovicova K, Vyletelova-Klimesova M, Babak V, Kalhotka L, Kolackova I, Karpiskova R (2016). Microbiological Quality of Raw Milk in the Czech Republic. Czech J Food Sci.

[bib1.bib9] Carloni E, Petruzzelli A, Amagliani G, Brandi G, Caverni F, Mangili P, Tonucci F (2016). Effect of farm characteristics and practices on hygienic quality of ovine raw milk used for artisan cheese production in central Italy. Anim Sci J.

[bib1.bib10] Claeys WL, Verraes C, Cardoen S, De Block J, Huyghebaert A, Raes K, Dewettinck K, Herman L (2014). Consumption of raw or heated milk from different species: an evaluation of the nutritional and potential health benefits. Food Contr.

[bib1.bib11] de Garnica ML, Linage B, Carriedo JA, De La Fuente LF, García-Jimeno MC, Santos JA,  Gonzalo C (2013). Relationship among specific bacterial counts and total bacterial and somatic cell counts and factors influencing their variation in ovine bulk tank milk. J Dairy Sci.

[bib1.bib12] Delavenne E, Mounier J, Asmani K, Jany JL, Barbier G, Le Blay G (2011). Fungal diversity in cow, goat and ewe milk. Int J of Food Microbiol.

[bib1.bib13] Duckova V, Cynikova M, Krocko M (2008). Enterokoky v potravinách živočišného pôvodu. (Enterococci in foods of animal origin – in the Slovak language). Mliekarstvo.

[bib1.bib14] Fotou K, Tzora A, Voidarou Ch, Alexopoulos A, Plessas S, Avgeris I, Bezirtzoglou E, Akrida-Demertzi K, Demertzis PG (2011). Isolation of microbial pathogens of subclinical mastitis from raw sheep's milk of Epirus (Greece) and their role in its hygiene. Anaerobe.

[bib1.bib15] Franz CH, Stiles ME, Schleifer KH, Holzapfel WH (2003). Enterococci in foods – a conundrum for food safety. Int J Food Microbiol.

[bib1.bib16] Gonzalo C (2017). Milk hygiene in small ruminants: A review. Span J Agri Res.

[bib1.bib17] Greifova M, Greif G, Leskova E, Meriova K (2003). Enterokoky – ich hodnotenie v mliekarenskej technológii. (Enterococci – their evaluation in dairy technology – in the Slovak language). Mliekarstvo.

[bib1.bib18] Jaramillo DP, Zamora A, Guamis B, Rodrigues M, Trujillo AJ (2008). Cheesemaking aptitude of two Spain dairy ewe breeds: Changes during lactation and relationships between physico-chemical and technological properties. Small Rumin Res.

[bib1.bib19] Kalhotka L, Dostalova L, Sustová K, Kuchtik J, Detvanova L (2015). Changes in the microflora composition of goat and sheep milk during lactation. Potravinárstvo.

[bib1.bib20] Khaldi Z, Nafti M, Jilani MT (2022). Small ruminants milk from Tunisian oasis: Physicochemical characteristics, mineral contents, and microbiological quality. Trop Anim Health Prod.

[bib1.bib21] Kondyli E, Svarnas C, Samelis J, Katsiari MC (2012). Chemical composition and microbiological quality of ewe and goat milk of native Greek breeds. Small Rumin Res.

[bib1.bib22] Koop G, Nielen M, Van Werven T (2009). Bulk milk somatic cell counts are related to bulk milk total bacterial counts and several herd-level risk factors in dairy goats. J Dairy Sci.

[bib1.bib23] Kuchtik J, Konecna L, Sykora V, Sustova K, Fajman M, Kos I (2017). Changes in physico-chemical characteristics, somatic cell count and curd quality during lactation and their relationships in Lacaune sheep. Mljekarstvo.

[bib1.bib24] Lacanin I, Duskova M, Kladnicka I, Karpiskova R (2015). Occurrence of *Enterococcus* spp. isolated from the milk and milk products. Potravinárstvo.

[bib1.bib25] Lebreton F, Willems RJL, Gilmore MS, Gilmore MS, Clewell DB, Ike Y, Shankar N (2014). Enterococci: From Commensals to Leading Causes of Drug Resistant Infection.

[bib1.bib26] Li S, Delger M, Dave A, Singh H, Ye A (2022). Seasonal variations in the composition and physicochemical characteristics of sheep and goat milks. Foods.

[bib1.bib27] Matutinovic S, Kalit S, Salajpal K, Vrdoljak J (2011). Effects of flock, year and season on the quality of milk from an indigenous breed in the sub-Mediterranean area. Small Rumin Res.

[bib1.bib28] Mayer HK, Fiechter G (2012). Physical and chemical characteristics of sheep and goat milk in Austria. Int Dairy J.

[bib1.bib29] Merlin Junior IA, Santos JS, Costa LG, Costa RG, Ludovico A, Rego FC, Santana EH (2015). Sheep milk: physical-chemical characteristics and microbiological quality. Arch Latinoam Nutr.

[bib1.bib30] Novotna L, Kuchtik J, Sustova K, Zapletal D, Filipcik R (2009). Effects of lactation stage and parity on milk yield, composition and properties of organic sheep milk. J Appl Anim Res.

[bib1.bib31] Paape MJ, Wiggans GR, Bannerman DD, Thomas D, Sanders AH, Contreras A, Moroni P, Miller RH (2007). Monitoring goat and sheep milk somatic cell counts. Small Rumin Res.

[bib1.bib32] Park YW, Albenzio MM, Sevi A, Haenlein GFW, Park YW, Haenlein G (2013). Milk and Dairy Products in Human Nutrition, Production, Composition and Health.

[bib1.bib33] Samarzija D, Zamberlin S, Pagacic T (2012). Psychrothropic bacteria and milk and dairy products quality. Mljekarstvo.

[bib1.bib34] Sevi A, Albenzio M, Taibi L, Dantone D, Massa S, Annicchiarico G (1999). Changes of somatic cell count through lactation and their effects on nutritional renneting and bacteriological characteristics of ewe's milk. Adv Food Sci.

[bib1.bib35] Silanikove N, Leitner G, Merin G, Prosser CG (2010). Recent advances in exploiting goat's milk: Quality, safety and production aspects. Small Rumin Res.

[bib1.bib36] Silva EOO, Nespolo CR, Pohl Sehn C, Cabral Pinheiro F, Stefani LM (2020). Lactic acid bacteria with antimicrobial, proteolytic and lipolytic activities isolated from ovine dairy products. Food Sci Technol Campinas.

[bib1.bib37] Švec P, Franz CHMAP, Holzapfel WH, Wood BJB (2014). The genus *Enterococcus*. Lactic Acid Bacteria: Biodiversity and Taxonomy.

[bib1.bib38] Tonamo A, Komlósi I, Varga L, Czeglédi L, Peles F (2020). Bacteriological quality of raw ovine milk from different sheep farms. Animals.

